# Fatigue in pediatric inflammatory bowel disease: Explained by transdiagnostic and disease‐focused factors

**DOI:** 10.1002/jpn3.70317

**Published:** 2025-12-23

**Authors:** Maartje D. Stutvoet, Anouk Vroegindeweij, Tessa Z. Toonen, Merel M. Nap‐Van der Vlist, Johanna W. Hoefnagels, Anemone van den Berg, Sanne L. Nijhof

**Affiliations:** ^1^ Department of Pediatrics, Wilhelmina Children's Hospital University Medical Center Utrecht, Utrecht University Utrecht the Netherlands; ^2^ Department of Pediatric Gastroenterology, Wilhelmina Children's Hospital University Medical Center Utrecht Utrecht the Netherlands

**Keywords:** child health, cohort studies, Crohn's disease, disease activity, ulcerative colitis

## Abstract

**Objective:**

Fatigue is highly prevalent in children with inflammatory bowel disease (IBD), even during clinical remission. This suggests a role for transdiagnostic factors—lifestyle, psychological, and social influences not specific to the disease. This study aimed to assess the prevalence of severe fatigue in pediatric IBD and evaluate its associations with both IBD‐focused and transdiagnostic factors.

**Methods:**

Children with IBD ages 8–18 from the PROactive cohort completed the Pediatric Quality of Life Inventory—Multidimensional Fatigue Scale. IBD‐focused clinical data were extracted from electronic health records. Transdiagnostic factors were assessed using validated patient‐reported outcome measures. Associations with fatigue were examined using linear regression.

**Results:**

Among 127 patients (mean age 14.9 ± 2.7 years; 43% male), most were in clinical remission. One hundred six patients self‐reported fatigue, and 99 parents reported their child′s fatigue. Severe fatigue was self‐reported by 29%. Of IBD‐focused factors, only disease activity (*β* = −0.40) and comorbidity (*β* = −0.18) showed significant associations with fatigue. Of transdiagnostic factors, lower physical, emotional, and social functioning; poorer sleep quality; less physical activity; more pain, anxiety, and depressive symptoms; lower life satisfaction and self‐rated health; and increased school absence and pressure were significantly associated with more fatigue. Backward selection retained only transdiagnostic factors in the multivariate model, which explained 78% of fatigue variance.

**Conclusion:**

Fatigue is common in children with IBD and is more strongly associated with transdiagnostic than disease‐focused factors. These findings highlight the importance of integrative care strategies that address modifiable psychosocial and lifestyle domains to reduce fatigue and improve functioning in children with IBD.

## Abstract (highlights)

What is Known

- Fatigue is a common, debilitating symptom across chronic childhood conditions, including inflammatory bowel disease.
- Previous research shows a limited association between fatigue and clinical disease activity.

What is New

- Transdiagnostic factors such as sleep quality, depressive symptoms, and social functioning account for most fatigue variance.
- Targeting modifiable transdiagnostic factors may be key to reducing fatigue and its effect on daily functioning.

## Graphical abstract

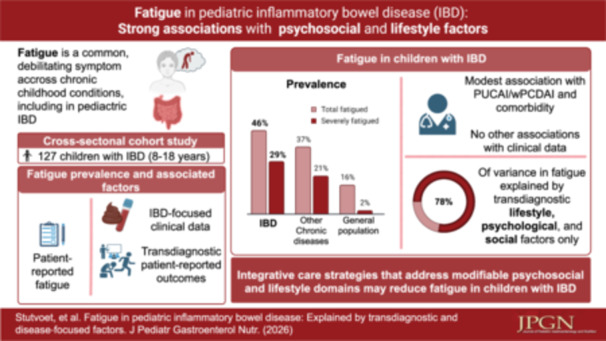

## 1 INTRODUCTION

Fatigue is a frequent and burdensome symptom in children with inflammatory bowel disease (IBD),^1^, ^2^ strongly linked to reduced quality of life.^3^, ^4^ Proposed mechanisms include subclinical inflammation, anemia, and sleep disturbances.^5^ Fatigue has been associated with higher disease activity,^3^, ^6^ older age,^3^ anxiety, depression,^7^, ^8^ and family dysfunction^8^ in pediatric IBD. Children, caregivers, and healthcare professionals have jointly identified fatigue as a top research priority,^9^ highlighting the need for better understanding and treatment.^1^, ^10^, ^11^, ^12^, ^13^

Fatigue is common across chronic pediatric conditions, such as other autoimmune diseases and childhood cancer.^14^, ^15^ Its widespread occurrence suggests that fatigue often stems from transdiagnostic factors—shared psychological, lifestyle, and social influences—rather than solely disease‐specific mechanisms.^14^, ^16^ Fatigue during clinical remission further supports this perspective.^1^, ^2^, ^4^, ^5^ It shows the need to assess beyond IBD‐focused clinical factors, specifically, to include transdiagnostic factors.

Reported fatigue prevalence in pediatric IBD varies widely (8%–23%),^4^, ^17^ likely due to inconsistent definitions and measurement tools, and cohort differences. Prior studies have often relied on disease‐specific measures^2^, ^4^ or lacked a comprehensive assessment.^1^ This study estimated the prevalence of severe fatigue using a validated, generic measure in a well‐characterized pediatric IBD cohort, and examined associations with both IBD‐focused and transdiagnostic factors. Understanding these associations may clarify whether IBD‐specific care is required, or whether integrative, transdiagnostic approaches addressing modifiable factors are more appropriate.^16^

## 2 METHODS

### 2.1 Ethics statement

The institutional review board classified the study as exempt from the Medical Research Involving Human Subjects Act (METC no. 16‐707/C). This study adheres to all local laws and the Declaration of Helsinki.

### 2.2 Study design and patients

This is a preregistered (DOI: 10.17605/OSF.IO/CK8J5) single‐center, cross‐sectional study in pediatric patients (ages 8–18) with IBD. All patients with IBD were selected from the PROactive cohort between October 2017 and September 2024.^18^ The PROactive cohort is a longitudinal study that collects transdiagnostic patient‐reported outcomes on fatigue, daily life participation, and psychosocial well‐being in children with chronic conditions. It is based at the Wilhelmina Children's Hospital in the Netherlands, an academic medical care center. Patients are generally invited 6–12 months after initial diagnosis, a timing that typically reflects a stable disease phase following completion of induction therapy. Before enrollment, patients and/or one of their parents provided informed consent to use clinical and patient‐reported data for research. Of the children approached for the PROactive IBD cohort, 127 (43%) provided informed consent and completed the questionnaires. Based on administrative records, the sample did not differ from nonparticipants in age. The most common reasons for nonparticipation were feeling preoccupied with participation in other research, no longer receiving care at the hospital, or unknown. More details about the PROactive cohort are described elsewhere.^18^

### 2.3 Data collection

The PROactive cohort collected transdiagnostic factors through patient and/or parent questionnaires administered via the online portal “www.hetklikt.nu” before an outpatient visit. For this cross‐sectional study, the earliest available time point with patient‐reported fatigue was selected. If patient‐reported fatigue was unavailable, the earliest time point with parent‐reported fatigue was used. IBD‐focused clinical data were extracted from electronic health records (EHRs).

### 2.4 Measurements

The primary outcome was fatigue, measured using the general fatigue subscale of the validated Dutch version of the Pediatric Quality of Life Inventory—Multidimensional Fatigue Scale (PedsQL‐MFS).^19^ This subscale has six items (e.g., “I feel tired,” “I feel too tired to do things that I like to do”) and yields a score ranging from 0 to 100, with lower scores indicating more fatigue.

IBD‐focused factors were selected to reflect disease characteristics such as disease activity. They were distinguished from transdiagnostic factors based on measures routinely used in IBD care. Factors indicating disease activity were fecal calprotectin (FC; ≥250 μg/g indicating active inflammation^20^), C‐reactive protein (CRP; ≥5 mg/L considered elevated^21^), and clinical disease activity. Clinical disease activity was based on the weighted Pediatric Crohn's Disease Activity Index (wPCDAI) for patients with Crohn's disease^22^ and the Pediatric Ulcerative Colitis Activity Index (PUCAI) for those with ulcerative colitis.^23^ Other IBD characteristics included age at and time since diagnosis, the presence of comorbidities (Supporting Information S1: Table S1), hemoglobin concentration (mmol/L), and current IBD medication use. Medication was categorized as biologicals (e.g., infliximab), immunosuppressants (e.g., azathioprine or methotrexate), and salicylates (e.g., mesalazine). All IBD‐focused factors were obtained from the EHR and matched to the questionnaire date as closely as possible, within a window of 3 months (Table 1).

**Table 1:** Patient characteristics.

	Mean ± SD, median [IQR] or *n* (%)
Number of participants	127
Male	55 (43)
Age (years)	14.9 ± 2.7
Age at diagnosis (years)	12.4 ± 3.5
Time since diagnosis (years)	1.5 [1.0; 3.2]
History of bowel surgery	5 (4)
IBD diagnosis	
Colitis ulcerosa	54 (43)
Crohn's disease	66 (52)
Unclassified	7 (6)
*Location according to Paris classification*	
UC/IBD‐U location (*n* = 61)	
E1: Ulcerative proctitis	7 (11)
E2: Left‐sided UC (distal to splenic flexure)	5 (8)
E3: Extensive UC (distal to hepatic flexure)	8 (13)
E4: Pancolitis	41 (67)
CD location (*n* = 66)	
L1: distal 1/3 ileum, limited cecal disease	12 (18)
L2: colonic	15 (23)
L3: ileocolonic	39 (59)
No upper tract involvement	46 (70)
Current medical treatment^a^	
Biologicals	59 (46)
Immunosuppressants	75 (59)
Salicylates	51 (40)
Clinical disease activity^b^	
PUCAI (*n* = 55)	5.0 [0.0; 12.5]
wPCDAI (*n* = 59)	10.0 [0.0; 21.25]
Clinical disease activity (*n* = 114)	
Remission	69 (61)
Mild	38 (33)
Moderate	6 (5)
Severe	1 (1)
Biochemical parameters^b^	
Fecal calprotectin ≥250 µg/g (*n* = 120)	23 (19)
CRP ≥ 5 mg/L (*n* = 114)	13 (11)
Hemoglobin (g/dL, *n* = 115)	8.42 ± 0.87
Anemia (*n* = 115)	14 (12)
Comorbidity^c^	
Auto‐immune	24 (19)
Persistent physical symptoms	14 (11)
Atopic	12 (9)
Neurological, developmental, and psychiatric	11 (9)
Other	22 (17)

*Note*: The number of participants is specified if it does not represent the full sample, due to missing data or characteristics relevant only to CD or UC.

Abbreviations: CD, Crohn's disease; CRP, C‐reactive protein; IBD‐U, inflammatory bowel disease unclassified; IQR, interquartile range; PUCAI, Pediatric Ulcerative Colitis Activity Index; SD, standard deviation; UC, ulcerative colitis; wPCDAI, weighted Pediatric Crohn's Disease Activity Index.

^a^ Totals exceed sample size because patients could use multiple medications.

^b^ Median days [IQR] between disease activity indicators and fatigue assessment: clinical disease acitivity 4 [1–12], fecal calprotectin 11 [6–23], CRP 8.5 [2–21], and hemoglobin 7 [1.5–21].

^c^ Supporting Information S1: Table S1 for comorbidities.

Transdiagnostic factors were assessed across lifestyle, psychological, and social domains, and were patient‐reported unless indicated otherwise. Physical, emotional, and social functioning were measured using the corresponding subscales of the PedsQL‐Generic Core Scales (GCS).^24^ Sleep/rest problems were assessed using the 6‐item “sleep/rest fatigue” subscale from the PedsQL‐MFS.^19^ All PedsQL subscales range from 0 to 100, with higher scores indicating better functioning/fewer difficulties. The PedsQL‐GCS and ‐MFS demonstrate good internal consistency, with Cronbach's *α* values ranging from 0.73 to 0.88 and 0.79 to 0.80, respectively.^19^, ^24^ Symptoms of anxiety and depression were measured using the corresponding subscales of the 25‐item Revised Child Anxiety and Depression Scale (RCADS‐25), which have good internal consistency. The anxiety and depression subscales range from 0 to 45 and 0 to 30, respectively, with higher scores indicating more severe symptoms.^25^ Perceived health, school pressure, and physical activity were evaluated using items from the Health Behavior in School‐aged Children questionnaire.^26^ Physical activity was measured by the number of days in the past week the patient was physically active, based on World Health Organization (WHO) recommendations,^27^ and has acceptable reliability.^28^ Average pain in the last week was indicated on a visual analog scale from 0 to 10.^29^ Obesity was defined as a body mass index (BMI) *z* score > 2 standard deviation (SD), calculated using normative data from the Third Dutch Growth study (1980) based on anthropometrics in the EHR.^30^ School absence, the only parent‐reported factor, was assessed by the percentage of school missed over the previous 6 months.

### 2.5 Data analysis

Patient and IBD characteristics, and fatigue scores were summarized using descriptive statistics. For normally distributed variables, we reported means and SDs, and for non‐normally distributed variables, medians, and interquartile ranges (IQRs). Fatigue and severe fatigue were respectively defined as a score >1 SD and >2 SDs below the normative mean, based on reference values from healthy Dutch controls matched for age and sex,^19^ factors known to influence fatigue.^31^ Thresholds for severe fatigue ranged from 43.3 to 62.0.^15^

To examine the associations between patient‐reported fatigue (continuous outcome) and IBD‐focused and transdiagnostic factors, we applied a stepwise analytic approach following multiple imputation of missing values in both outcome and predictor variables using the MICE package in R (MICE package, R).^32^ The proportion of missing data ranged from 0% (e.g., medication use, age) to 23% for school absence (Supporting Information S1: Table S2).

First, we conducted exploratory Spearman's rank correlations to identify candidate factors (*p* < 0.05). Second, candidate factors were entered into separate linear regression models adjusted for age and sex. Third, we constructed a comprehensive multivariable model to explain fatigue variance using backward selection, beginning with variables showing *p* < 0.01 in the adjusted linear regressions. All assumptions for linear regression were verified. To evaluate the robustness of our findings, we performed a sensitivity analysis using the original (nonimputed) dataset.

## 3 RESULTS

The mean age of the 127 participating patients was 14.9 ± 2.7 years, and 43% were male (Table 1). Most were diagnosed with Crohn's disease (52%, *n* = 66), followed by ulcerative colitis (42%, *n* = 54) and unclassified IBD (6%, *n* = 7). The median time since diagnosis was 1.5 years IQR [1.0; 3.2]. The majority were on immunosuppressants (59%, *n* = 75), and a substantial proportion on biologicals (46%, *n* = 51). Nearly all patients were in clinical remission: of the seven patients (6%) with moderate to severe clinical disease activity, only three (3%) had elevated FC levels (≥250 µg/g) as well. In total, elevated FC levels were observed in 23 patients (19%).

### 3.1 Fatigue

**Figure 1:**
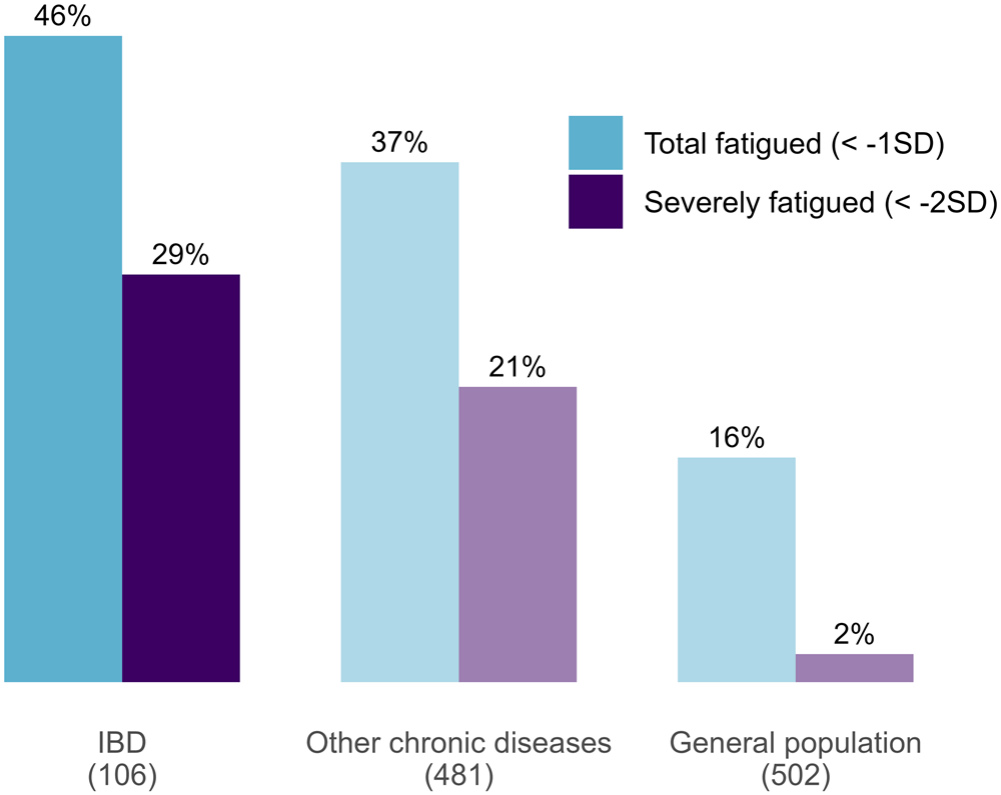
Fatigue prevalence in pediatric IBD. For reference, fatigue prevalence measured using the same “general fatigue” subscale of the MFS‐GCS is also shown for other chronic childhood diseases^15^ and the general Dutch population.^19^ Sample sizes are indicated. Fatigue prevalence in IBD was based on patient self‐report. GCS, Generic Core Scales; IBD, inflammatory bowel disease; MFS, Multidimensional Fatigue Scale; SD, standard deviation.

Patient‐reported fatigue was available for 106 patients, and parent‐reported fatigue for 99 patients: 86 patients had both. Almost half of the patients reported fatigue (46%, *n* = 49), with a substantial proportion experiencing severe fatigue (29%, *n* = 31) (Figure 1). Parent‐reported fatigue and severe fatigue prevalences were similar, at 45% (*n* = 45) and 26% (*n* = 26), respectively. The mean patient‐reported general fatigue score was 65 ± 25, with a corresponding *z* score of –0.97 ± 1.83.

### 3.2 Association of IBD‐focused factors with fatigue

Of the IBD‐focused factors significantly correlated with patient‐reported fatigue (Table 2), higher clinical disease activity (*β* = −0.40, *p* < 0.01) and the presence of comorbidity (*β* = −0.18, *p* = 0.048) remained significantly associated with more fatigue in the linear regression model adjusted for age and sex (Table 3). In contrast, age at diagnosis (*p* = 0.087) and time since diagnosis (*p* = 0.084) were no longer significantly associated with fatigue after adjustment. No other IBD‐focused factors showed significant associations with fatigue (Table 2).

**Table 2:** IBD‐focused correlates of fatigue (*n* = 127).

Associated factors	*r*	*p*	FMI^a^
Age at diagnosis (years)	−0.20	0.03	0.07
Time since diagnosis (years)	0.20	0.03	0.08
Crohn's disease (yes)	0.05	0.61	0.07
Biologicals (yes)	−0.07	0.46	0.04
Immunosuppressants (yes)	0.08	0.41	0.09
Salicylates (yes)	−0.12	0.22	0.10
Clinical disease activity (PUCAI/wPCDAI; “remission” to “moderate to severe”)^b^	−0.40	<0.001	0.19
Fecal calprotectin ≥ 250 µg/g (yes)	0.00	0.98	0.13
CRP ≥ 5 mg/L (yes)	−0.09	0.38	0.18
Hemoglobin (g/dL)	0.08	0.41	0.05
Comorbidity (yes)	−0.20	0.04	0.11

*Note*: Spearman's correlations; fatigue (patient‐reported, generic subscale PedsQL‐MFS, 0–100); negative *r* indicates more fatigue.

Abbreviations: CRP, C‐reactive protein; FMI, fraction of missing information; IBD, inflammatory bowel disease; MFS, Multidimensional Fatigue Scale; PedsQL, Pediatric Quality of Life Inventory; PUCAI, Pediatric Ulcerative Colitis Activity Index; wPCDAI, weighted Pediatric Crohn's Disease Activity Index.

^a^ Proportion of uncertainty attributable to missing data.

^b^ The levels moderate and severe were merged to account for the low prevalence of severe clinical disease activity.

**Table 3:** Integrative overview of IBD‐focused and transdiagnostic associations with fatigue (*n* = 127).

	Univariate				Multivariate^b^ (adj. *R* ^2^ = 78%)		
Associated factor	*β*	Unst. *β*	95% CI	FMI^a^	*β*	Unst. *β*	95% CI
IBD‐focused factors							
Age at diagnosis (years)	−0.23	−1.6	−3.4; 0.2	0.06			
Time since diagnosis (years)	0.15	1.6	−0.2; 3.4	0.04			
Clinical disease activity (PUCAI/wPCDAI; “remission” to “moderate to severe”)^c^	−0.40	−15.6	−22.6; −8.7**	0.19			
Comorbidity (yes)	−0.18	−8.8	−17.5; −0.1*	0.11			
Transdiagnostic biological/lifestyle factors							
Pain (NRS, 0–10)	−0.48	−4.2	−5.7; −2.6**	0.26			
Physically active days/week (0–7)	0.21	2.4	0.1; 4.6*	0.29			
Sleep/rest quality (PedsQL‐MFS subscale; 0–100)	0.61	0.8	0.6; 1.1**	0.27	0.19	0.3	0.1; 0.4**
Self‐rated health (0–3; “Excellent” to “Bad”)	−0.60	−17.2	−21.9; −12.5**	0.25	−0.13	−3.7	−8.6; 1.1
Physical functioning (PedsQL‐GCS subscale; 0–100)	0.71	1.0	0.8; 1.2**	0.25	0.24	0.3	0.1; 0.6**
Transdiagnostic psychological factors							
Anxiety symptoms (subscale RCADS; 0–45)	−0.38	−2.0	−2.8; −1.0**	0.20	0.20	1.1	0.3; 1.8**
Depression symptoms (subscale RCADS; 0–30)	−0.75	−3.9	−4.5; −3.2**	0.19	−0.50	−2.7	−3.5; −1.8**
Life satisfaction (Cantril Ladder; 0–10)	0.47	7.0	4.4; 9.7**	0.20	−0.05	−0.8	−2.9; 1.3
Emotional functioning (PedsQL‐GCS; 0–100)	0.48	0.6	0.4; 0.8**	0.15			
Transdiagnostic social factors							
School absence (%)	−0.52	−0.5	−0.6; −0.3**	0.18	−0.06	−0.1	−0.2; 0.1
School pressure (0–3, “not at all” to “a lot”)	−0.30	−8.2	−13.9; −2.4**	0.36	−0.08	−2.2	−5.7; 1.4
Social functioning (PedsQL‐GCS subscale; 0–100)	0.57	0.9	0.7; 1.1**	0.13	0.12	0.2	−0.0; 0.4

*Note*: Linear regression models adjusted for age and sex; dependent variable fatigue (patient‐reported, generic subscale PedsQL‐MFS, 0–100); negative *β* indicates more fatigue.

Abbreviations: CI, confidence interval; FMI, fraction of missing information; GCS, Generic Core Scales; IBD, inflammatory bowel disease; MFS, Multidimensional Fatigue Scale; NRS, numeric rating scale; PedsQL, Pediatric Quality of Life Inventory; PUCAI, Pediatric Ulcerative Colitis Activity Index; RCADS, Revised Child Anxiety and Depression Scale; SD, standard deviation; unst., unstandardized; wPCDAI, weighted Pediatric Crohn's Disease Activity Index.

a Proportion of uncertainty attributable to missing data.

b Backward model included factors associated with fatigue (*p* < 0.01) in univariate analyses. The final model had the highest *R* ^2^.

c The levels moderate and severe were merged to account for the low prevalence of severe clinical disease activity.

* *p* < 0.05;

** *p* < 0.01.

### 3.3 Association of transdiagnostic factors with fatigue

Obesity was the only transdiagnostic factor not significantly correlated with patient‐reported fatigue (*r* = −0.052, *p* = 0.58). In linear regression models adjusted for the age and sex, lower fatigue levels were significantly associated (*p* < 0.001 unless indicated otherwise) with better physical (*β* = 0.71), social (*β* = 0.57), and emotional functioning (*β* = 0.48), better sleep/rest quality (*β* = 0.61), higher life satisfaction (*β* = 0.47) and more days of physical activity (*β* = 0.21, *p* = 0.040). Conversely, higher fatigue was significantly associated with more depressive (*β* = −0.75) and anxiety symptoms (*β* = −0.38), poorer self‐rated health (*β* = −0.60), more school absence (*β* = −0.52) and school pressure (*β* = −0.30, *p* = 0.006), and more pain (*β* = −0.48) (Table 3).

### 3.4 Full explanatory model

The final multivariable model explained 78% of the variance in fatigue (Table 3). It initially included all factors significantly associated with fatigue in univariable analyses (*p* < 0.01), adjusted for age and sex. Emotional functioning was excluded due to multicollinearity with depressive symptoms, and clinical disease activity and pain were removed during backward selection because their exclusion improved model fit. The resulting model retained only transdiagnostic factors. Depressive symptoms remained the strongest predictor (*β* = −0.50), while associations with most other factors were small. The direction of the association between anxiety symptoms and fatigue changed from negative in the univariable analysis to positive in the multivariable model. Post hoc inspection revealed no evidence of multicollinearity or suppression effects to explain this reversal.

### 3.5 Sensitivity analyses

Descriptive statistics were similar for original and imputed data (Supporting Information S1: Table S2). Characteristics also aligned between those with and without patient‐reported fatigue data (*n* = 21, Supporting Information S1: Table S3). Patients with missing IBD‐focused factors (*n* = 24) were less often male (21% vs. 49%, *p* = 0.025, Supporting Information S1: Table S4). Repeating analyses on the original data showed consistent directions of associations (Supporting Information S1: Tables S5 and S6). Univariate models showed no changes in direction or significance (Supporting Information S1: Table S6). In the multivariable model (*n* = 74), directions were stable with two small shifts in significance.

## 4 DISCUSSION

This study demonstrates that fatigue is highly prevalent in pediatric IBD, with 29% of patients reporting severe fatigue—despite most being in clinical remission. While clinical disease activity showed a modest univariable association, only transdiagnostic factors explained unique variance in the final model (78%).

The prevalence of severe fatigue in our pediatric IBD cohort (29%) was substantially higher than in the general population (2%).^19^ It also appears slightly higher than in other chronic pediatric diseases (21%) (Figure 1),^15^ as well as a previous Dutch IBD cohort (23%).^4^ While these findings show a notable burden of fatigue in pediatric IBD, the apparent differences with other chronic diseases and the earlier IBD study should be interpreted with caution, given the relatively small sample sizes of both IBD studies. Direct comparisons across diagnoses are further complicated by the absence of a shared disease activity metric, though shared patterns in transdiagnostic associations suggest possible common mechanisms.^16^ A potential difference with the previous IBD cohort could be attributable to differences in setting (only academic vs. also nonacademic) and the inclusion of patients regardless of disease activity. Notably, the mean general fatigue score in our cohort fell within the range reported in earlier studies (60.87–75.30).^3^, ^33^ Importantly, our findings confirm the presence of fatigue also during clinical remission,^3^, ^4^ a pattern also observed in other pediatric autoimmune diseases.^14^, ^15^ While our cross‐sectional design limits conclusions about the chronicity of fatigue, the persistence of fatigue in adults with IBD over time^34^ underscores the importance of addressing fatigue in children to mitigate long‐term impacts.

In our study, among the IBD‐focused factors, only clinical disease activity was significantly associated with fatigue in univariate analysis (*p* < 0.01). This is consistent with earlier studies showing modest associations between clinical disease activity and fatigue^2^, ^3^, ^4^, ^33^, ^34^, ^35^ and no association with biochemical markers such as FC, CRP, and anemia.^2^, ^3^, ^4^, ^11^, ^35^ The discrepancy between clinical and biological markers may reflect the inclusion of functional limitations in the clinical disease activity scores, and their weak correlation with measures of biological disease activity as FC.^36^ It should be noted that IBD subtype‐specific associations with IBD‐focused factors may exist,^35^ and we were unable to include all potentially relevant factors, such as prior bowel surgery and being underweight, due to limited variability. Additionally, our sample consisted largely of patients in clinical remission, which may have reduced the sensitivity to detect associations with biological disease severity. Yet, the lack of associations with objective disease markers mirrors findings in other pediatric conditions.^14^, ^16^ Taken together, the association with clinical disease activity shows that fatigue may be closely linked to perceived disease‐related limitations rather than inflammatory activity alone.

Transdiagnostic factors—including depressive symptoms, poor sleep, and social functioning—were strongly associated with fatigue, consistent with previous findings in pediatric IBD cohorts,^3^, ^4^, ^33^ and other chronic diseases.^14^, ^16^ Interestingly, the inverse association between anxiety and fatigue in our multivariable model contrasts with adult IBD studies, where higher anxiety has been linked to greater fatigue.^11^ However, a similar inverse trend has been reported in other chronic childhood diseases,^16^ though the mechanism remains unclear. It is possible that contextual factors, such as support from parents and friends, need to be taken into account to properly interpret this association.

Our results emphasize the complex interactions among psychosocial factors and support a more integrative approach to fatigue assessment. This aligns with the biopsychosocial model of fatigue, which proposes that fatigue arises from dynamic interactions among biological, psychological, and social contributors.^37^ While disease activity may initially precipitate fatigue, other factors—such as poor sleep or school pressure—can perpetuate it, even after inflammation resolves.^38^ Although our cross‐sectional design precludes causal inference, the consistent associations across lifestyle, psychological, and social domains—including school absence and reduced quality of life—underscore the broad impact of fatigue and suggest that these factors may also contribute to its persistence. The prominence of transdiagnostic factors in our findings highlights their potential as modifiable intervention targets, particularly in patients in clinical remission.

Currently, no evidence‐based treatments for fatigue in pediatric IBD exist.^39^ However, modifiable factors such as sleep, physical activity, and depressive symptoms represent promising targets. These are transdiagnostic factors on which children with IBD do not necessarily perform worse than healthy peers.^4^, ^40^, ^41^ Yet, these factors have been associated with fatigue in IBD before,^4^, ^5^, ^11^ and have been shown to longitudinally predict fatigue in adults with IBD,^34^ in the general pediatric population,^42^, ^43^, ^44^ and in children postcancer.^45^ Given that these modifiable factors are not IBD‐specific, non‐IBD‐specific interventions—such as cognitive behavioral therapy and graded exercise—may be relevant in managing fatigue in pediatric IBD.^46^, ^47^

Our study has several limitations. First, the representativeness of our study sample is uncertain due to the low PROactive participation rate among the IBD population at our center and limited information on nonparticipants. Additionally, as the study was conducted in an academic hospital, the findings may not generalize to other clinical settings. Retrospective EHR‐based collection of disease activity indicators could have introduced bias if disease activity was highly fluctuating. A major impact is unlikely, given the short median 4‐ to 8.5‐day interval with the fatigue assessment, which reflects the preceding 2 weeks. Regarding model construction, we selected the final model based on adjusted *R* ^2^ rather than formal model fit comparisons. While a more parsimonious model might be preferable for clinical application, we aimed to provide a broader overview of fatigue‐associated factors. Nevertheless, given the sample size and data limitations, our overview is not exhaustive and could benefit from including additional constructs, such as family dysfunction.^8^ Also, post hoc analyses were not feasible due to sample size. Finally, the cross‐sectional design precludes conclusions about causality. Future longitudinal research is needed to identify children most at risk for persistent fatigue and determine optimal timing for intervention.

On the other hand, this study has strengths. We used a validated fatigue questionnaire with reference values from the Dutch adolescent population, allowing for comparisons with both healthy peers and children with other chronic diseases.^15^ Using generic instruments for assessing transdiagnostic constructs, rather than IBD‐specific quality of life measures, enhances comparability across conditions. Focusing on the general fatigue subscale of the PedsQL‐MFS allowed for distinguishing fatigue from sleep quality. Finally, our reliance on patient‐reported outcomes highlights the lived experiences of children with IBD, recognizing the well‐established discrepancy between child self‐report and parent proxy‐report.^48^

## 5 CONCLUSION

Fatigue is common in pediatric IBD, even during clinical remission. IBD‐focused clinical factors, reflecting disease severity and activity, showed no or modest associations with fatigue, and did not contribute unique variance in the multivariate model. In contrast, strong associations with transdiagnostic factors such as depressive symptoms, sleep quality, and quality of life underscore the need to routinely monitor not only fatigue but also related psychosocial and lifestyle domains. Based on child self‐reports, our findings highlight the relevance of the patient perspective and support an integrative health model that extends beyond IBD‐focused follow‐up. In the absence of active disease, targeting modifiable transdiagnostic factors may be key to reducing fatigue and its impact. Parallels with other chronic pediatric diseases suggest that interventions developed for other contexts may also benefit children with IBD. Longitudinal research is needed to identify which children are at risk for persistent fatigue, when to intervene, and how transdiagnostic interventions can effectively improve fatigue‐related outcomes.

## CONFLICT OF INTEREST STATEMENT

The authors declare no conflicts of interest.

## Supporting information

2025‐12‐10_IBD_fatigue_Supplementary_Clean.

## References

[1] Van de Vijver E , Van Gils A , Beckers L , Van Driessche Y , Moes ND , van Rheenen PF . Fatigue in children and adolescents with inflammatory bowel disease. World J Gastroenterol. 2019;25(5):632‐643. 30774277 10.3748/wjg.v25.i5.632 PMC6371006

[2] Turner ST , Focht G , Orlanski‐Meyer E , et al. Fatigue in pediatric inflammatory bowel diseases: a systematic review and a single center experience. J Pediatr Gastroenterol Nutr. 2024;78(2):241‐251. 38374545 10.1002/jpn3.12039

[3] Marcus SB , Strople JA , Neighbors K , et al. Fatigue and Health‐Related quality of life in pediatric inflammatory bowel disease. Clin Gastroenterol Hepatol. 2009;7(5):554‐561. 19418604 10.1016/j.cgh.2009.01.022

[4] Bevers N , Van de Vijver E , Hanssen A , et al. Fatigue and physical activity patterns in children with inflammatory bowel disease. J Pediatr Gastroenterol Nutr. 2023;77(5):628‐633. 37494540 10.1097/MPG.0000000000003905

[5] Borren NZ , van der Woude CJ , Ananthakrishnan AN . Fatigue in IBD: epidemiology pathophysiology and management. Nat Rev Gastroenterol Hepatol. 2019;16(4):247‐259. 30531816 10.1038/s41575-018-0091-9

[6] Pirinen T , Kolho KL , Simola P , Ashorn M , Aronen ET . Parent and self‐report of sleep‐problems and daytime tiredness among adolescents with inflammatory bowel disease and their population‐based controls. Sleep. 2010;33(11):1487‐1493. 21102990 10.1093/sleep/33.11.1487 PMC2954698

[7] Ondersma SJ , Lumley MA , Corlis ME , Tojek UM , Tolia V . Adolescents with inflammatory bowel disease: the roles of negative affectivity and hostility in subjective versus objective health. J Pediatr Psychol. 1997;22(5):723‐738. 9383932 10.1093/jpepsy/22.5.723

[8] Tojek TM , Lumley MA , Corlis M , Ondersma S , Tolia V . Maternal correlates of health status in adolescents with inflammatory bowel disease. J Psychosom Res. 2002;52:173‐179. 11897236 10.1016/s0022-3999(01)00291-4

[9] Crohn & Colitis NL. Kinderen met darmziekten maken Top 10 Onderzoeksagenda [Children with intestinal diseases create a Top 10 Research Agenda] [Internet]. 2023. Accessed June 21, 2023. https://www.crohn-colitis.nl/blog/actueel/kinderen-met-darmziekten-maken-top-10-onderzoeksagenda/

[10] Hindryckx P , Laukens D , D'Amico F , Danese S . Unmet needs in IBD: the case of fatigue. Clin Rev Allergy Immunol. 2018;55(3):368‐378. 28852978 10.1007/s12016-017-8641-4

[11] D'Silva A , Fox DE , Nasser Y , et al. Prevalence and risk factors for fatigue in adults with inflammatory bowel disease: a systematic review with meta‐analysis. Clin Gastroenterol Hepatol. 2022;20(5):995‐1009.e7. 34216824 10.1016/j.cgh.2021.06.034

[12] Czuber‐Dochan W , Norton C , Bredin F , Darvell M , Nathan I , Terry H . Healthcare professionals' perceptions of fatigue experienced by people with IBD. J Crohns Colitis. 2014;8(8):835‐844. 24491516 10.1016/j.crohns.2014.01.004

[13] Nocerino A , Nguyen A , Agrawal M , Mone A , K, Swaminath , A, Lakhani . Fatigue in inflammatory bowel diseases: etiologies and management. Adv Ther. 2020;37:97‐112. 31760611 10.1007/s12325-019-01151-w PMC6979464

[14] Williams K , Loades M . How common is fatigue across chronic health conditions in children and young people? A systematic review. Open Rev J (online). 2023;8:1‐29.

[15] Nap‐Van Der Vlist MM , Dalmeijer GW , Grootenhuis MA , et al. Fatigue in childhood chronic disease. Arch Dis Child. 2019;104(11):1090‐1095. 31175124 10.1136/archdischild-2019-316782

[16] Nap‐Van Der Vlist MM , Dalmeijer GW , Grootenhuis MA , et al. Fatigue among children with a chronic disease: a cross‐sectional study. BMJ Paediatr Open. 2021;5(1):e000958. 10.1136/bmjpo-2020-000958 PMC7893660 33665374

[17] Schuchard J , Carle AC , Kappelman MD , Tucker CA , Forrest CB , et al. Interpreting patient‐reported outcome scores: pediatric inflammatory bowel disease as a use case. Acad Pediatr. 2022;22(8):1520‐1528. 34995822 10.1016/j.acap.2021.12.029 PMC9253201

[18] Nap‐ van der Vlist MM , Hoefnagels JW , Dalmeijer GW , et al. The PROactive cohort study: rationale, design, and study procedures. Eur J Epidemiol. 2022;37(9):993‐1002. 35980506 10.1007/s10654-022-00889-y PMC9385417

[19] Gordijn SM , Cremers EMP , Kaspers GJL , Gemke RJBJ . Fatigue in children: reliability and validity of the Dutch PedsQL^TM^ multidimensional fatigue scale. Qual Life Res. 2011;20(7):1103‐1108. 21246290 10.1007/s11136-010-9836-9 PMC3161196

[20] Van Rheenen PF , Aloi M , Assa A , et al. The medical management of paediatric Crohn's disease: an ECCO‐ESPGHAN guideline update. J Crohns Colitis. 2021;15(2):171‐194. 10.1093/ecco-jcc/jjaa161 33026087

[21] Turner D , Ricciuto A , Lewis A , et al. STRIDE‐II: an update on the Selecting Therapeutic Targets in Inflammatory Bowel Disease (STRIDE) Initiative of the International Organization for the Study of IBD (IOIBD): determining therapeutic goals for treat‐to‐target strategies in IBD. Gastroenterology. 2021;160(5):1570‐1583. 33359090 10.1053/j.gastro.2020.12.031

[22] Turner D , Levine A , Walters TD , et al. Which PCDAI version best reflects intestinal inflammation in pediatric crohn disease? J Pediatr Gastroenterol Nutr. 2017;64(2):254‐260. 27050050 10.1097/MPG.0000000000001227

[23] Turner D , Otley AR , Mack D , et al. Development, validation, and evaluation of a pediatric ulcerative colitis activity index: a prospective multicenter study. Gastroenterology. 2007;133(2):423‐432. 17681163 10.1053/j.gastro.2007.05.029

[24] Engelen V , Haentjens MM , Detmar SB , Koopman HM , Grootenhuis MA . Health related quality of life of Dutch children: psychometric properties of the PedsQL in the Netherlands. BMC Pediatr. 2009;9:68. 19887000 10.1186/1471-2431-9-68 PMC2776579

[25] Ebesutani C , Reise SP , Chorpita BF , et al. The Revised Child Anxiety and Depression Scale‐Short Version: scale reduction via exploratory bifactor modeling of the broad anxiety factor. Psychol Assess. 2012;24(4):833‐845. 22329531 10.1037/a0027283

[26] Currie C, Zanotti C, Morgan A, et al. *Social Determinants of Health and Well‐being Among Young People*. Vol. 6, Health Policy for Children and Adolescents. World Health Organization Regional Office for Europe; 2012.

[27] World Health Organization . Global Recommendations on Physical Activity for Health. World Health Organization; 2010. 26180873

[28] Ng K , Hämylä R , Tynjälä J , et al. Test‐retest reliability of adolescents' self‐reported physical activity item in two consecutive surveys. Arch Public Health. 2019;77:91‐98. 10.1186/s13690-019-0335-3 PMC6388478 30891238

[29] Rosier EM , Iadarola MJ , Coghill RC . Reproducibility of pain measurement and pain perception. Pain. 2002;98:205‐216. 12098633 10.1016/s0304-3959(02)00048-9

[30] Roede MJ . Growth diagrams 1980. Netherlands third national survey. Tijdsch Soc Gezondh. 1980;63:1‐34.

[31] Ter Wolbeek M , Van Doornen LJP , Kavelaars A , Heijnen CJ . Severe fatigue in adolescents: a common phenomenon? Pediatrics. 2006;117(6):e1078‐e1086. 16740810 10.1542/peds.2005-2575

[32] Buuren S , Groothuis‐Oudshoorn K . Mice: multivariate imputation by chained equations in R. J Stat Softw. 2011;45(3):1‐67.

[33] Zuo Y , Cao J , Wang Y , Cai W , Li M . Fatigue in children and adolescents with inflammatory bowel disease: a cross‐sectional study. Front Pediatr. 2024;12:1519779. 39748815 10.3389/fped.2024.1519779 PMC11693721

[34] Borren NZ , Long MD , Sandler RS , Ananthakrishnan AN . Longitudinal trajectory of fatigue in patients with inflammatory bowel disease: a prospective study. Inflamm Bowel Dis. 2021;27(11):1740‐1746. 33367749 10.1093/ibd/izaa338 PMC8528141

[35] Holten KA , Bernklev T , Opheim R , et al. Fatigue in patients with inflammatory bowel disease in remission one year after diagnosis (the IBSEN III Study). J Crohns Colitis. 2025;19(4):jjae170. 39527064 10.1093/ecco-jcc/jjae170 PMC12001332

[36] Hoekman DR , Diederen K , Koot BGP , Tabbers MM , Kindermann A , Benninga MA . Relationship of clinical symptoms with biomarkers of inflammation in pediatric inflammatory bowel disease. Eur J Pediatr. 2016;175(10):1335‐1342. 27573259 10.1007/s00431-016-2762-2 PMC5031739

[37] Geenen R , Dures E . A biopsychosocial network model of fatigue in rheumatoid arthritis: a systematic review. Rheumatology. 2019;58(suppl 5):v10‐v21. 31682275 10.1093/rheumatology/kez403 PMC6827269

[38] Lievesley K , Rimes KA , Chalder T . A review of the predisposing, precipitating and perpetuating factors in chronic fatigue syndrome in children and adolescents. Clin Psychol Rev. 2014;34(3):233‐248. 24632047 10.1016/j.cpr.2014.02.002

[39] Farrell D , Artom M , Czuber‐Dochan W , Jelsness‐Jørgensen LP , Norton C , Savage E . Interventions for fatigue in inflammatory bowel disease. Cochrane Database Syst Rev. 2020;4(4):CD012005. 32297974 10.1002/14651858.CD012005.pub2 PMC7161727

[40] Moorman EL , Koskela‐Staples NC , Janicke DM . A systematic review of sleep disturbances in pediatric inflammatory bowel disease. J Pediatr Psychol. 2023;48(3):267‐282. 36688543 10.1093/jpepsy/jsac088

[41] Stapersma L , van den Brink G , Szigethy EM , Escher JC , Utens EMWJ . Systematic review with meta‐analysis: anxiety and depression in children and adolescents with inflammatory bowel disease. Aliment Pharmacol Ther. 2018;48(5):496‐506. 29984495 10.1111/apt.14865

[42] Ter Wolbeek M , Van Doornen LJP , Kavelaars A , Heijnen CJ . Predictors of persistent and new‐onset fatigue in adolescent girls. Pediatrics. 2008;121(3):e449‐e457. 18310166 10.1542/peds.2007-1093

[43] Rimes KA , Goodman R , Hotopf M , Wessely S , Meltzer H , Chalder T . Incidence, prognosis, and risk factors for fatigue and chronic fatigue syndrome in adolescents: a prospective community study. Pediatrics. 2007;119(3):e603‐e609. 17332180 10.1542/peds.2006-2231

[44] Collin SM , Norris T , Deere KC , Jago R , Ness AR , Crawley E . Physical activity at age 11 years and chronic disabling fatigue at ages 13 and 16 years in a UK birth cohort. Arch Dis Child. 2018;103(6):586‐591. 29382635 10.1136/archdischild-2017-314138 PMC5965358

[45] Van Dijk‐Lokkart EM , Steur LMH , Braam KI , et al. Longitudinal development of cancer‐related fatigue and physical activity in childhood cancer patients. Pediatr Blood Cancer. 2019;66(12):e27949. 31436372 10.1002/pbc.27949

[46] Hulme K , Safari R , Thomas S , et al. Fatigue interventions in long term, physical health conditions: a scoping review of systematic reviews. PLoS One. 2018;13:e0203367. 30312325 10.1371/journal.pone.0203367 PMC6193578

[47] Higson‐Sweeney N , Mikkola A , Smith L , et al. Nonpharmacological interventions for treating fatigue in adolescents: a systematic review and narrative synthesis of randomised controlled trials. J Psychosom Res. 2022;163:111070. 36327529 10.1016/j.jpsychores.2022.111070

[48] Rostagno E , Marchetti A , Bergadano A , et al. Concordance between paediatric self‐reports and parent proxy reports on fatigue: a multicentre prospective longitudinal study. Eur J Oncol Nurs. 2020;49:101829. 33120214 10.1016/j.ejon.2020.101829

