# Supplementary material for: Fatigue in pediatric inflammatory bowel disease: Explained by transdiagnostic and disease‐focused factors
Source: J Pediatr Gastroenterol Nutr. 2025 Dec 23;82(3):699–707. doi: 10.1002/jpn3.70317 (PMC12964513; doi:10.1002/jpn3.70317)
Supplement: Supplementary file 1 — 2025‐12‐10_IBD_fatigue_Supplementary_Clean. [file JPN3-82-699-s001.docx]

| **Supplementary Table 1** Included comorbidities per category | |
| --- | --- |
| **Category** | **Diagnoses** |
| **Autoimmune** | Henoch–Schönlein purpura, Psoriasis, Autoimmune sclerosing cholangitis, Juvenile Idiopathic Arthritis, IBD-related arthritis, Erythema nodosum, Coeliac disease, Autoimmune hepatitis, Autoimmune thyroiditis, Neutrophilic dermatosis, Immunodeficiency (e.g., Common Variable Immunodeficiency), Immunoglobulin A deficiency. |
| **Persistent physical symptoms** | Chronic pain syndrome, Irritable bowel syndrome, Chronic fatigue syndrome, Autonomic dysregulation, Chronic back and headache complaints, Functional abdominal pain, Pain syndrome. |
| **Atopic** | Eczema (constitutional, seborrheic, medication-induced), Allergies (e.g., to nuts, milk, fruit, inhalation allergens), Anaphylaxis (e.g., to infliximab), Asthma, Hypereosinophilia. |
| **Neurological, developmental & ssychiatric** | Idiopathic intracranial hypertension, Attention-deficit/hyperactivity disorder, Anxiety disorder, Self-harm, Avoidant/Restrictive Food Intake Disorder, Autism Spectrum Disorder, Rolandic epilepsy. |
| **Other** | Prematurity (e.g., 34 weeks), Intensive Care Unit admission for bilateral pneumonia, Urinary incontinence after valve resection, Gilbert’s syndrome, Constipation, Steroid-induced hypertension, Heterozygous Beta Thalassemia, Exocrine pancreatic insufficiency due to pancreatitis, Hereditary spherocytosis, Secondary amenorrhea. |

| **Supplementary Table 2** Summary statistics of variables of interest. Patient-reported fatigue (PedsQL-MFS) was missing in 17% of participants, with the highest proportion of missing data observed for school absence (23%). No specific patterns of missingness were identified. CRP and FC were log-transformed prior to imputation to approximate normal distributions and subsequently back-transformed to create binary variables for analysis. In addition to the variables of interest, the imputation model included parent-reported scores on the PedsQL-MFS (22% missing) and PedsQL-GCS (21% missing), as well as patient-reported scores on the CIS-4 fatigue scale (13% missing). An overview of the percentage of missing data for each variable included in the analysis is presented. Multiple imputation was conducted using predictive mean matching, generating 25 imputed datasets with 40 iterations each. Convergence was confirmed through diagnostic checks: all R̂ (R-hat) values were below 1.2, and trace plots indicated adequate mixing and stability. | | | |
| --- | --- | --- | --- |
| **Variables in analyses** | **Original**  **(non-imputed)** | **Missing** | **Imputed** |
|  | Mean ± SD, median [IQR] or n (%) | (%) | Mean ± SD, median [IQR] or n (%) |
| General fatigue (PedsQL-MFS subscale; 0–100) | 65.2 (24.8) | 17 | 65.7 (24.2) |
| Sex (male) | 43.3% | 0 | 43.3% |
| Age (years) | 14.9 (2.7) | 0 | 14.9 (2.7) |
| Age at diagnosis (years) | 12.3 (3.5) | 0 | 12.3 (3.5) |
| Time since diagnosis (years) | 1.5 [2.2] | 0 | 1.5 [2.2] |
| Crohn's disease (yes) | 52% | 0 | 52% |
| Biologicals (yes) | 46.5% | 0 | 46.5% |
| Immunosuppressants (yes) | 59.1% | 0 | 59.1% |
| Salicylates (yes) | 40.2% | 0 | 40.2% |
| Clinical disease activity (PUCAI/wPCDAI; 'remission (0)' to 'moderate to severe') | 0: 60.5% | 10 | 0: 59.1% |
| Fecal calprotectin ≥ 250 µg/g (yes) | 19.2% | 6 | 19.7% |
| CRP ≥5 mg/L (yes) | 11.4% | 10 | 12.6% |
| Hemoglobine (g/dL) | 8.4 (0.9) | 9 | 8.4 (0.9) |
| Comorbidity (yes) | 51.2% | 0 | 51.2% |
| Obesity (BMI > 2SD, yes) | 10.8% | 6 | 11% |
| Pain (NRS, 0-10) | 3.3 (2.8) | 15 | 3.3 (2.8) |
| Physically active days/week (0-7) | 3.8 (2.1) | 17 | 3.7 (2.2) |
| Sleep/rest quality (PedsQL-MFS subscale; 0–100) | 63.0 (17.4) | 17 | 63.1 (17.6) |
| Self-rated health (0-3; 'Excellent' to 'Bad') | 1: 40.6%; 2: 37.7% | 17 | 1: 39.4%; 2: 37% |
| Physical functioning (PedsQL-GCS subscale; 0–100) | 79.2 (17.8) | 10 | 79.1 (17.7) |
| Anxiety symptoms (subscale RCADS; 0 - 45 ) | 5.1 (4.6) | 16 | 5.3 (4.7) |
| Depression symptoms (subscale RCADS; 0 - 30) | 6.0 (4.6) | 16 | 6.1 (4.6) |
| Life satisfaction (Cantril Ladder; 0-10) | 7.1 (1.6) | 17 | 7.1 (1.6) |
| Emotional functioning (PedsQL-GCS; 0–100) | 74.2 (18.9) | 10 | 74.1 (18.9) |
| School absence (%) | 5.0 [19.0] | 23 | 5.1 [19.0] |
| School pressure (0-3, ‘not at all’ to ‘a lot’) | 1: 51%; 2: 15.4% | 18 | 1: 46.5%; 2: 17.3%; |
| Social functioning (PedsQL-GCS subscale; 0–100) | 85.4 (15.3) | 10 | 85.1 (15.3) |
| Abbreviations: adj. is adjusted, BMI is body mass index, CRP is C-reactive protein, GSC is Generic Core Scales, MFS is Multidimensional Fatigue Scale, NRS is numeric rating scale, PedsQL is Pediatric Quality of Life Inventory, PUCAI is Pediatric Ulcerative Colitis Activity Index, RCADS is Revised Child Anxiety and Depression Scale, unst. is unstandardized, wPCDAI is weighted Pediatric Crohn’s Disease Activity Index. | | | |

| **Supplementary Table 3** Comparison of patients with and without patient-reported fatigue | | | | |
| --- | --- | --- | --- | --- |
|  | **Total** | **Patient-reported fatigue available** | **Patient-reported fatigue missing** | **p^a^** |
|  | Mean ± SD, median [IQR] or n (%) | | |  |
| Number of participants | 127 | 106 | 21 |  |
| Male | 55 (43.3) | 46 (43.4) | 9 (42.9) | 1.00 |
| Age (years) | 14.87 (2.70) | 14.96 (2.62) | 14.43 (3.10) | 0.42 |
| Age at diagnosis (years) | 12.35 (3.54) | 12.42 (3.48) | 12.00 (3.87) | 0.62 |
| Crohn's disease (yes) | 66 (52.0) | 55 (51.9) | 11 (52.4) | 1.00 |
| Biologicals (yes) | 59 (46.5) | 47 (44.3) | 12 (57.1) | 0.40 |
| Immunosuppressants (yes) | 75 (59.1) | 62 (58.5) | 13 (61.9) | 0.96 |
| Salicylates (yes) | 51 (40.2) | 42 (39.6) | 9 (42.9) | 0.97 |
| PUCAI | 5.00 [0.00, 12.50] | 5.00 [0.00, 15.00] | 0.00 [0.00, 10.00] | 0.68 |
| wPCDAI | 10.00 [0.00, 21.25] | 10.00 [0.00, 27.50] | 0.00 [0.00, 9.38] | 0.06 |
| Clinical disease activity |  |  |  | 0.78 |
| Remission | 69 (60.5) | 55 (58.5) | 14 (70.0) |  |
| Mild | 38 (33.3) | 33 (35.1) | 5 (25.0) |  |
| Moderate | 6 (5.3) | 5 (5.3) | 1 (5.0) |  |
| Severe | 1 (0.9) | 1 (1.1) | 0 (0.0) |  |
| Fecal calprotectin ≥ 250 µg/g (yes) | 23 (19.2) | 18 (18.0) | 5 (25.0) | 0.68 |
| CRP ≥5 mg/L (yes) | 13 (11.4) | 10 (10.6) | 3 (15.0) | 0.87 |
| Hemoglobine (g/dL) | 8.42 (0.87) | 8.42 (0.92) | 8.42 (0.57) | 0.99 |
| Comorbidity (yes) | 65 (51.2) | 55 (51.9) | 10 (47.6) | 0.91 |
| **^a^**For continuous variables, we used independent t-tests for normally distributed data and the Wilcoxon rank-sum test for non-normally distributed data. For categorical variables, we employed χ² tests or Fisher's exact test when expected cell counts in any group were below five.  Abbreviations: CRP is C-reactive protein, PUCAI is Pediatric Ulcerative Colitis Activity Index, wPCDAI is weighted Pediatric Crohn’s Disease Activity Index. | | | | |

| **Supplementary Table 4** Comparison of patients with and without full data on IBD-focused factors | | | | |
| --- | --- | --- | --- | --- |
|  | **Total** | **Full data on IBD-focused factors** | **Missing data on IBD-focused factors** | **p^a^** |
|  | Mean ± SD, median [IQR] or n (%) | | |  |
| Number of participants | 127 | 103 | 24 |  |
| Male | 55 (43.3) | 50 (48.5) | 5 (20.8) | 0.03 |
| Age (years) | 14.87 (2.70) | 14.73 (2.76) | 15.45 (2.39) | 0.24 |
| Age at diagnosis (years) | 12.35 (3.54) | 12.24 (3.55) | 12.81 (3.53) | 0.49 |
| Crohn's disease (yes) | 66 (52.0) | 55 (53.4) | 11 (45.8) | 0.66 |
| Biologicals (yes) | 59 (46.5) | 50 (48.5) | 9 (37.5) | 0.45 |
| Immunosuppressants (yes) | 75 (59.1) | 63 (61.2) | 12 (50.0) | 0.44 |
| Salicylates (yes) | 51 (40.2) | 38 (36.9) | 13 (54.2) | 0.19 |
| PUCAI | 5.00 [0.00, 12.50] | 5.00 [0.00, 15.00] | 0.00 [0.00, 2.50] | 0.17 |
| wPCDAI | 10.00 [0.00, 21.25] | 10.00 [0.00, 20.00] | 25.00 [10.00, 41.88] | 0.09 |
| Clinical disease activity |  |  |  | 0.66 |
| Remission | 69 (60.5) | 61 (59.2) | 8 (72.7) |  |
| Mild | 38 (33.3) | 36 (35.0) | 2 (18.2) |  |
| Moderate | 6 (5.3) | 5 (4.9) | 1 (9.1) |  |
| Severe | 1 (0.9) | 1 (1.0) | 0 (0.0) |  |
| Fecal calprotectin ≥ 250 µg/g (yes) | 23 (19.2) | 19 (18.4) | 4 (23.5) | 0.87 |
| CRP ≥5 mg/L (yes) | 13 (11.4) | 11 (10.7) | 2 (18.2) | 0.81 |
| Hemoglobine (g/dL) | 8.42 (0.87) | 8.43 (0.87) | 8.33 (0.93) | 0.72 |
| Comorbidity (yes) | 65 (51.2) | 52 (50.5) | 13 (54.2) | 0.92 |
| **^a^**For continuous variables, we used independent t-tests for normally distributed data and the Wilcoxon rank-sum test for non-normally distributed data. For categorical variables, we employed χ² tests or Fisher's exact test when expected cell counts in any group were below five.  Abbreviations: CRP is C-reactive protein, PUCAI is Pediatric Ulcerative Colitis Activity Index, wPCDAI is weighted Pediatric Crohn’s Disease Activity Index. | | | | |

| **Supplementary Table 5** IBD-focused correlates of fatigue in non-imputed data | | | |
| --- | --- | --- | --- |
|  | **n** | **r** | **p** |
| Age at diagnosis (years) | 106 | -0.23 | 0.02 |
| Time since diagnosis (years) | 106 | 0.26 | 0.01 |
| Crohn's diesease (yes) | 106 | 0.03 | 0.79 |
| Biologicals (yes) | 106 | -0.04 | 0.71 |
| Immunosuppressants (yes) | 106 | 0.09 | 0.37 |
| Salicylates (yes) | 106 | -0.12 | 0.22 |
| Clinical disease activity (PUCAI/wPCDAI; 'remission' to 'moderate to severe') | 94 | -0.42 | <0.001 |
| Fecal calprotectin ≥ 250 µg/g (yes) | 100 | -0.01 | 0.93 |
| CRP ≥5 mg/L (yes) | 94 | -0.18 | 0.09 |
| Hemoglobine (g/dL) | 95 | 0.13 | 0.22 |
| Comorbidity (yes) | 106 | -0.24 | 0.01 |
| Obesity (BMI > 2 SD, yes) | 101 | 0.13 | 0.19 |
| Pain (NRS, 0-10) | 103 | -0.53 | <0.001 |
| Physically active days/week (0-7) | 97 | 0.25 | 0.02 |
| Sleep/rest quality (PedsQL-MFS subscale; 0–100) | 106 | 0.65 | <0.001 |
| Self-rated health (0-3; 'Excellent' to 'Bad') | 97 | -0.64 | <0.001 |
| Physical functioning (PedsQL-GCS subscale; 0–100) | 106 | 0.81 | <0.001 |
| Anxiety symptoms (subscale RCADS; 0 - ) | 101 | -0.41 | <0.001 |
| Depression symptoms (subscale RCADS; 0 - ) | 101 | -0.77 | <0.001 |
| Life satisfaction (Cantril Ladder; 0-10) | 97 | 0.45 | <0.001 |
| Emotional functioning (PedsQL-GCS; 0–100) | 106 | 0.58 | <0.001 |
| School absence (%) | 84 | -0.56 | <0.001 |
| School pressure (0-3, ‘not at all’ to ‘a lot’) | 95 | -0.35 | 0.001 |
| Social functioning (PedsQL-GCS subscale; 0–100) | 106 | 0.69 | <0.001 |
| Spearman’s correlations, patients reported fatigue using the generic subscale of the PedsQL-Multidimensional Fatigue Scale (range 0-100). A negative r indicates more fatigue.  Abbreviations: adj. is adjusted, BMI is body mass index, CRP is C-reactive protein, GSC is Generic Core Scales, n is number of observations, NRS is numeric rating scale, PedsQL is Pediatric Quality of Life Inventory, PUCAI is Pediatric Ulcerative Colitis Activity Index, RCADS is Revised Child Anxiety and Depression Scale, unst. is unstandardized, wPCDAI is weighted Pediatric Crohn’s Disease Activity Index. | | | |

| **Supplementary Table 6** Integrative overview of IBD-focused and transdiagnostic associations with fatigue in non-imputed data | | | | |
| --- | --- | --- | --- | --- |
|  | **n** | **β** | **Unst. β** | **95% CI** |
| *IBD-focused factors* |  |  |  |  |
| Age at diagnosis (years) | 106 | -0.27 | -1.92 | -3.9; 0.1 |
| Time since diagnosis (years) | 106 | 0.18 | 1.92 | -0.1;3.9 |
| Clinical disease activity (PUCAI/wPCDAI; 'remission' to 'moderate to severe') | 94 | -0.41 | -16.77 | -24.1; -9.4** |
| Comorbidity (yes) | 106 | -0.21 | -10.56 | -19.9; -1.2* |
| *Transdiagnostic biological/lifestyle factors* | | |  |  |
| Pain (NRS, 0-10) | 103 | -0.49 | -4.42 | -6.0; -2.9** |
| Physically active days/week (0-7) | 97 | 0.25 | 2.96 | 0.7; 5.2** |
| Sleep/rest quality (PedsQL-MFS subscale; 0–100) | 106 | 0.65 | 0.93 | 0.7; 1.1** |
| Self-rated health (0-3; 'Excellent' to 'Bad') | 97 | -0.64 | -19.12 | -23.6; -14.6** |
| Physical functioning (PedsQL-GCS subscale; 0–100) | 106 | 0.73 | 1.02 | 0.8; 1.2** |
| *Transdiagnostic psychological factors* | | |  |  |
| Anxiety symptoms (subscale RCADS; 0 - 45 ) | 101 | -0.39 | -2.11 | -3.1; -1.1** |
| Depression symptoms (subscale RCADS; 0 - 30 ) | 101 | -0.77 | -4.17 | -4.8; -3.5** |
| Life satisfaction (Cantril Ladder; 0-10) | 97 | 0.51 | 7.94 | 5.1; 10.8** |
| Emotional functioning (PedsQL-GCS; 0–100) | 106 | 0.54 | 0.71 | 0.5; 0.9** |
| *Transdiagnostic social factors* |  |  |  |  |
| School absence (%) | 84 | -0.56 | -0.59 | -0.8; -0.4** |
| School pressure (0-3, ‘not at all’ to ‘a lot’) | 95 | -0.34 | -9.89 | -15.3; -4.4** |
| Social functioning (PedsQL-GCS subscale; 0–100) | 106 | 0.62 | 1.01 | 0.8; 1.2** |
| Linear regression models adjusted for age and sex. Dependent variable fatigue, patient-reported using the generic subscale of the PedsQL-Multidimensional Fatigue Scale (range 0-100). A negative β indicates more fatigue. *p-value < 0.05, **p-value < 0.01 Abbreviations: CI is confidence interval, FMI is fraction of missing information, GSC is Generic Core Scales, n is number of observations, NRS is numeric rating scale, PedsQL is Pediatric Quality of Life Inventory, PUCAI is Pediatric Ulcerative Colitis Activity Index, SD is standard deviation, RCADS is Revised Child Anxiety and Depression Scale, unst. is unstandardized, wPCDAI is weighted Pediatric Crohn’s Disease Activity Index | | | | |
